# Notes on Our Visits to Colleges and Hospitals in Philadelphia

**Published:** 1872-07

**Authors:** 


### NOTES ON OUR VISIT TO COLLEGES AND HOSPITALS IN PHILADELPHIA.

After the adjournment of the Association, the writer visited University of Pennsylvania—being an alumnus of that institution. Twenty-six years have passed since, with diploma in hand, we left this noble institution. As we stood before it, with years of toil and trials intervening between the past and the present, memory brought vividly to view the days of our student life. The "old mother" has changed but little—time has made but sorry work in defacing the grand old building. As we looked upon it, thoughts of the old time came rushing upon the brain, and with mingled feelings of sadness and pleasure, we reviewed the past. But while many familiar objects greeted the eye, the revered faces of many old teachers were not there to bid us welcome. Others, distinguished and gifted, now fill their places, and the old institution remains as ever second to none in the land.

We met Drs. Agnew and Rogers, who kindly showed us through the building. In the clinical lecture rooms we were introduced to Drs. Strawbridge, Duhring and Morris, whom we found busily engaged in attending to patients. Much information can be daily acquired by students visiting these clinics—so ably conducted by these young but accomplished scientific gentlemen.

We then visited the Anatomical Museum. It has been greatly increased in value. It looked as familiar as in 1854 and '56, when the writer was a student. It may be interesting to our readers—all of whom are doubtless familiar with the character of the old and honored institution—to give a brief sketch of the University. Its foundation was laid by the widow of the late Professor Wistar. She purchased and presented in 1818 to the University his collection, which was added to by the bequest of the late Professor W. E. Horner, in 1854. The clinical cabinet of splendid anatomical and pathological preparations—the result of the patient toil of an entire life-time of this truly good and great man, Professor Horner—was valued at $10,000. Models of wax, made by Dr. Chovet, and in plaster casts and crayon drawings, which were originally sent to this country by Dr. Fathengell of London, in 1762, were added to this collection, and include a very large number of interesting specimens of morbid anatomy, and of minute structure, besides preparations in natural history, and in comparative anatomy numerous drawings and also models. It has been an object to keep seperate the wet and dry preparations in their localities and classification. The first section contains models, paintings and books. Among these may be seen the model of a Chinese foot, the bust of Esculapius, the portraits of Professors Sheffeen and the trepanning instruments used by Dr. Wistar. The series marked A. consist of wet preparations, the fibrine of the blood, Protiene and Albumin dried, and uric acid.

*2nd Section*—Bones: Their Diseases and Accidents. Os humeri and teeth of a pig colored with madder; Callus after fracture of the bones of the leg; Caries in spine of infant; mollities of os femoris.

*3d Section*—Organs of Circulation. Adult and infant heart; Aneurism of thoracic Aorta; Ossification of the valves of the Aorta; Hypertrophy of heart; Varicose aneurism of the femoral artery.

*4th Section*—Organs of Respiration; Larynx and trachea injected; Fœtal lung; Adult do.; trachea with membrane of croup; collapsed lung from empyema; apoplexy of lung.

*5th Section*—Organs of Digestion. Teeth of pig colored by madder; do. upon a board; do. with the nerves; Pharynx and Esophagus; stomach minutely injected; Peyers glands in ilium section of small intestines injected; cancer of the tongue; hemorrhoidal tumor; liver abscess; schirrus liver.

*6th Section*—Emunctories. Kidney injected minutely; kidneys natural; coalition of, across spine; fungus hæmatodes of kidney; sugar of diabetes mellitus.

*7th Section*—Male Organs of Generation. Tubuli semiviferi unrevillid; testicle injected with quicksilver; prostate gland enlarged; stricture of urethra, calculi weight 1⅝ oz; elephantiasis of scrotum; carcimoma of testis.

*8th Section*—Female Organs of Generation. Female organs of generation with hymen; uterus injected; uterus virgin; ovarian graffian vescicles of fallopean tube; dropsy of ovarium; schirrus of the uterus; ovaria, dropsy on both sides.

*9th Section*—Conception and pregnancy; uterus two days after delivery; placenta adhering to uterus; placenta injected minutely; decidua reflexa with chorion and amnion; ovum of six weeks; embryo of ten weeks; uterus in third month of pregnancy.

*10th Section*—Organs of Sensation. Encephalon; spinal marrow; nerves; cerebrum injected minutely; cerebellum; spinal marrow of adult; arachnoid membrane of medulla spinalis.

*11th Section*—Embraces the eye, ear, nose, skin, touch and miscellaneous articles in morbid anatomy.

The above is an imperfect and meagre description of the most valuable museum in this country.

It will be seen that the student will have excellent advantages at this great medical centre of learning, of a practical and useful character. We will speak at greater length of these advantages when we come to notice the hospitals of Philadelphia.

We also visited the Jefferson Medical College, a description of which we must defer (for want of space) for our next issue.

